# Food substances alter gut resistome: Mechanisms, health impacts, and food components

**DOI:** 10.1111/1541-4337.70143

**Published:** 2025-03-06

**Authors:** Ze Liang, Zijian Liang, Hang‐Wei Hu, Kate Howell, Zhongxiang Fang, Pangzhen Zhang

**Affiliations:** ^1^ School of Agriculture, Food and Ecosystem Sciences, Faculty of Science The University of Melbourne Parkville Victoria Australia

**Keywords:** antibiotic resistance, dietary components, gut resistome, health

## Abstract

Antibiotics are effective in treating bacterial infections, but their widespread use has spurred antibiotic resistance, which is linked closely with human disease. While dietary components are known to influence the gut microbiome, specific effects on the gut resistome—the collection of antibiotic‐resistant genes in the gut—remain underexplored. This review outlines the mechanisms of antibiotic action and the development of resistance, emphasizing the connection between the gut resistome and human diseases such as metabolic disorders, cardiovascular disease, liver disease, and nervous system disorders. It also discusses the effects of diet habits and dietary components, including bioactive macronutrients, phytochemicals, and probiotics, on the composition of the gut resistome by enhancing antibiotic efficacy and potentially reducing resistance. This review highlights the emerging trend of increasing interest in functional foods aimed at targeting the gut resistome and a growing focus on bioactive plant compounds with the potential to modulate antibiotic resistance.

## 1 INTRODUCTION

Over 100 trillion microorganisms live in the human gut, collectively referred to as the gut microbiota. These microorganisms, including bacteria, viruses, fungi, and archaea, play an essential role in digestion, metabolism, and immune health (Lamberte & van Schaik, 2022; Valdes et al., 2018). Diverse species live in the human gut with representatives from Firmicutes, Bacteroidetes, Proteobacteria, Actinobacteria, Fusobacteria, and Verrucomicrobia phyla. Among these, bacteria from the phyla Firmicutes and Bacteroidetes account for over 90% of the population (Magne et al., 2020). However, the composition of gut microbiota varies significantly among individuals due to diet, genetics, and environment. The imbalance in gut microbiota, known as dysbiosis, has been linked to various diseases, including cognitive disorders and nervous system disorders (Quan et al., 2023). Achieving a balanced microbial community in the gut is therefore essential for maintaining overall health and reducing the risks of diseases.

In healthy adults, the gut microbiota establish a symbiotic relationship with the human hosts, maintaining homeostasis of the immune and nervous system and stabilizing the metabolic health of the host (Fan & Pedersen, 2021). A healthy gut microbiota is regarded as a stable and diverse community of microorganisms, yet it can be profoundly perturbed when exposed to systemic antibiotics (Lee et al., 2023). Global antibiotic usage reached 15.2 defined daily doses per 1000 population per day in 2023, and poor application, treatment, and abuse promote the development of antibiotic resistance (Klein et al., 2024). Such practices accelerate the development of antibiotic resistance, a process whereby bacteria evolve mechanisms to survive exposure to antibiotics. Although antibiotic resistance has occurred naturally long before the Anthropocene, human activities, including the improper disposal and excessive use of antibiotics, have amplified the problem (Hernandez et al., 2012).

Antibiotic resistance genes (ARGs) are prevalent in the human gut microbiota residing in strictly anaerobic gut commensals (Li et al., 2019). Gut resistome, which refers to the collection of all the ARGs present in the gut microbiota, can be obtained from the natural environment and food intake and can further accumulate with selection pressures induced by antibiotics and other antimicrobial substances in food (Casals‐Pascual et al., 2018). ARGs can be inherited from parent bacteria (vertical gene transfer) or acquired from other microorganisms through horizontal gene transfer, a process where genetic material moves between bacteria, even across species (Patangia et al., 2022). The enrichment of the gut resistome not only exacerbates antibiotic resistance but also contributes to the emergence of multidrug‐resistant infections (Zhang et al., 2022). These infections pose a significant public health challenge, as they are more difficult to treat, often requiring more costly therapies that may have greater side effects and be less effective (Ahmed et al., 2024).

Dietary substances are known to affect the incidence, activity, and diversity of the human gut microbiome. The types of nutrients consumed can either promote the growth of beneficial bacteria or lead to an imbalance that may contribute to health issues (Rinninella et al., 2023). For example, research shows that the Mediterranean diet (high intake of whole, unprocessed plant foods, olive oil, and dairy; moderate amounts of poultry and fish; and minimal consumption of red meat) is linked to an increase in *Faecalibacterium* spp. and short‐chain fatty acids (SCFAs), which have anti‐inflammatory effects. In contrast, a Western diet (high in calorie and rich in animal protein, saturated fats, simple sugars, and ultraprocessed foods, while being low in fiber, fruits, and vegetables) is associated with a higher abundance of *Blautia*, *Bacteroides*, and *Ruminococcus* species, which is linked to an increased risk of metabolic disorders and chronic inflammation (Ross et al., 2024). Building on this, diet not only influences beneficial bacteria but also has the potential to alter the presence of antibiotic‐resistant bacteria in the gut (Vliex et al., 2024). So far, bioactive macronutrients, phytochemicals, and probiotics in food have been found to alter antibiotic‐resistant bacteria in the gut, opening a new research window on functional food for modulation of gut resistome (Stege et al., 2022).

Despite the increasing body of literature exploring the link between diet, gut microbiota, and antibiotic resistance, the specific role of dietary components in modulating the gut resistome remains underexplored. While several reviews have discussed the impact of diet on the microbiome and antibiotic resistance, many focus on either broad dietary patterns or specific bacteria, with limited emphasis on the molecular mechanisms by which food‐derived bioactive compounds influence the gut resistome. Additionally, the potential of functional foods—those that target the gut resistome through modulation of microbial communities and antibiotic resistance—has not been fully investigated. This review aims to fill these gaps by providing a comprehensive analysis of the acquisition and accumulation of the gut resistome, its connection to human health, and the modulatory effects of dietary components, particularly phytochemicals, on the resistome. We propose a framework for future research focused on dietary interventions to limit the acquisition and development of antibiotic resistance in humans and the food chain and outline the foundations for developing functional foods that can effectively modulate the gut resistome.

## 2 OCCURRENCE AND TRANSMISSION OF ANTIBIOTIC RESISTANCE

Antibiotic resistance is a critical global health concern, arising from the ability of antibiotics to fight against bacterial infections by inhibiting or killing bacteria (Ramirez et al., 2020). When antibiotics are medically applied, the population and genetic profile of gut microbial communities may be altered and the risk of antibiotic resistance accumulation in the gut is increased. Here, we focus on antibiotics that target bacteria, summarizing their mechanisms of action and discussing how antibiotic resistance develops and accumulates in the gut.

### 2.1 The mechanism by which antibiotics control bacteria

**FIGURE 1:**
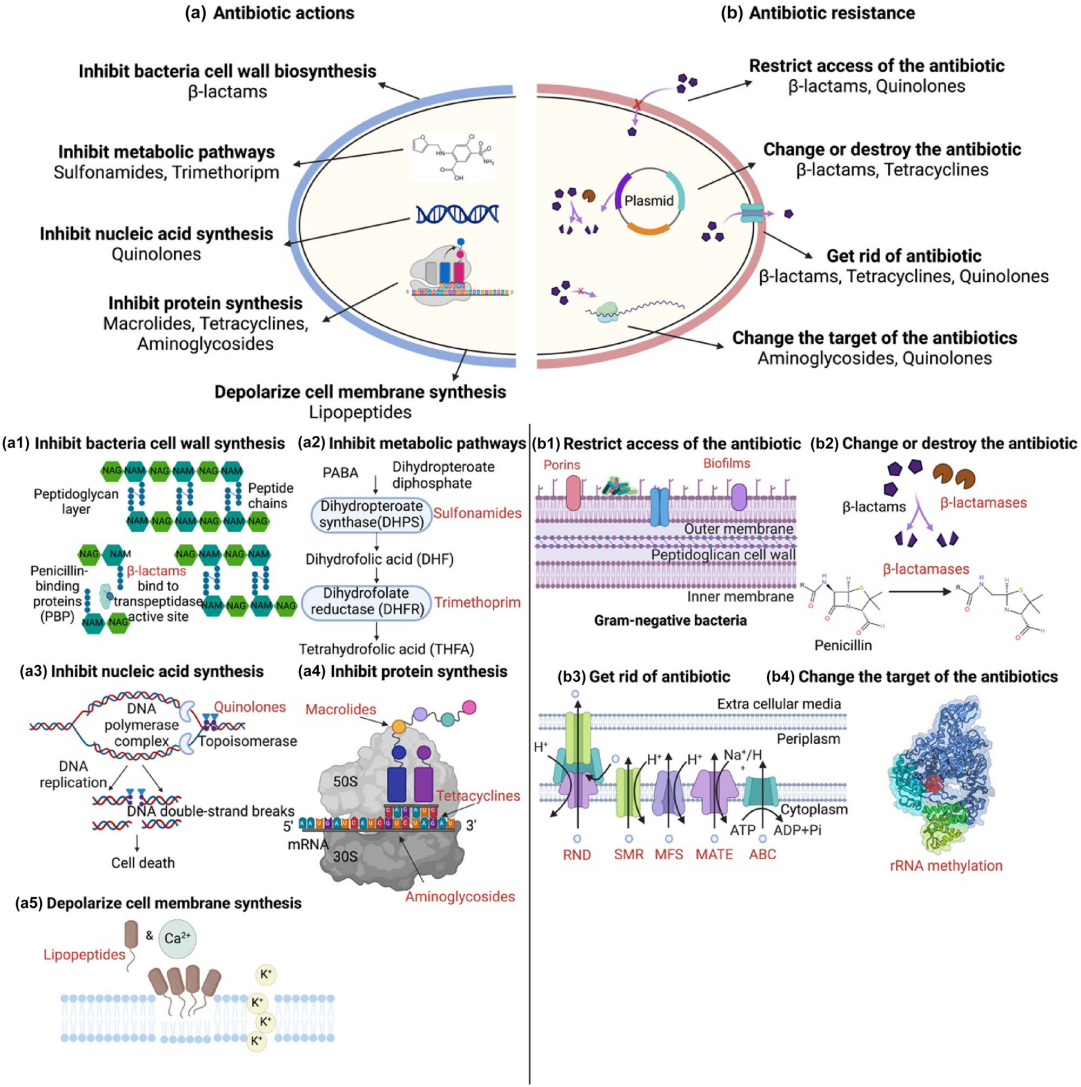
Mechanisms of antibiotic actions and antibiotic resistance. (a) Antibiotic actions: Antibiotics target key bacterial processes to inhibit growth and survival. (a1) β‐Lactams bind to transpeptidase, weaken the bacterial cell wall, and prevent the formation of peptidoglycan cross‐links. (a2) Sulfonamides and trimethoprim disrupt tetrahydrofolic acid synthesis and block nucleotide synthesis and DNA production. (a3) Quinolones inhibit bacterial RNA polymerase and topoisomerase enzymes, preventing DNA supercoiling and replication. (a4) Macrolides, tetracyclines, and aminoglycosides affect ribosomes and halt translation. (a5) Lipopeptides disrupt bacterial cell membranes by forming pores that cause depolarization. (b) Antibiotic resistance: Bacteria develop resistance through various strategies. (b1) Bacteria restrict antibiotic access through changes in the outer membrane that block entry or by forming biofilms that create physical barriers, reducing antibiotic penetration and efficacy. (b2) Bacteria produce β‐lactamases to degrade β‐lactams, rendering them ineffective by preventing their ability to bind to target proteins. (b3) Efflux pumps (ATP binding cassette [ABC], small multidrug resistance [SMR], multidrug and toxin extrusion [MATE], major facilitator superfamily [MFS], and resistance nodulation cell division [RND]) remove antibiotics from bacterial cells, reducing intracellular antibiotic concentration. (b4) Bacteria alter the target binding sites, such as through 16S rRNA methyltransferases that modify ribosomal RNA, reducing the binding efficiency of aminoglycosides and thereby diminishing their effectiveness.

Introduction of antibiotics to treat bacterial disease into clinical application was the greatest medical breakthrough in the 20th century (Hutchings et al., 2019). The antimicrobial mechanisms of antibiotics can be subdivided into five categories: inhibition of bacterial cell wall biosynthesis, interfering with metabolic pathways, disrupting nucleic acid synthesis, inhibiting protein synthesis, and depolarizing cellular membranes (Figure 1).

Specific antibiotics impede the synthesis of bacterial cell walls, leading to bacterial cell ruptures and apoptosis (Figure 1, a1). For instance, β‐lactam antibiotics are compounds with a β‐lactam ring in their chemical structure, giving a similar structure to d‐alanyl‐d‐alanine, which is the substrate for DD‐transpeptidase, also known as endogenous penicillin‐binding proteins. The structural similarities allow antibiotics to bind to DD‐transpeptidase and acetylate its serine residues at the active site. This prevents DD‐transpeptidase from further binding with its natural substrate to form key hydrogen bonds at the binding site. As a result, this would inhibit the final transpeptidation step and disrupt the cross‐linking of peptidoglycan chains to form the peptidoglycan layer of bacterial cell walls (Pancu et al., 2021). Without appropriate cross‐linking, the peptidoglycan framework weakens, compromising the cell wall's structural integrity and ultimately leading to cell death. Typical β‐lactam antibiotics include penicillins, cephalosporins, carbapenems, and monobactams.

Some antibiotics can interfere with specific enzymes or molecules involved in key metabolic reactions, effectively impeding the bacterium's ability to generate energy or synthesize essential molecules, thereby arresting their growth and replication (Figure 1, a2). Sulfonamide antibiotics are synthetic antimicrobial drugs that contain the sulfonamide group (Ovung & Bhattacharyya, 2021). Sulfonamides inhibit dihydropteroate synthase, an essential enzyme in the production of folate. This inhibition blocks the synthesis of nucleotides, which are necessary for DNA and RNA production, preventing bacterial replication and leading to cell death (Sathiyaseelan et al., 2021). Sulfamethoxydiazine, sulfamethazine, sulfamethoxazole, and sulfadiazine are the most used common sulfonamide antibiotics (Jansomboon et al., 2016). Likewise, another antibiotic, trimethoprim targets dihydrofolate reductase, an enzyme involved in the synthesis of folic acid, ultimately hindering the production of bacterial DNA. Trimethoprim can be used alone or together with sulfonamides, as it acts on a subsequent enzymatic step within the same pathway as sulfonamides, disrupting bacterial folate metabolism (Skold, 2010).

Another mechanism of antibiotic action is the inhibition of bacterial RNA polymerase required for the biosynthesis of nucleic acid, thereby preventing cell division and growth (Figure 1, a3). Quinolone antibiotics hinder DNA supercoiling and relaxation by binding to both DNA and gyrase, stabilizing the gyrase‐DNA‐cleaved complex. These antibiotics also inhibit nucleic acid synthesis by trapping the topoisomerase on the DNA, preventing it from supercoiling or relaxing the DNA (Correia et al., 2017). By inhibiting these enzymes, quinolone antibiotics prevent the separation of DNA strands, ultimately resulting in the formation of lethal DNA double‐strand breaks and inhibiting the growth of bacteria. Norfloxacin, ciprofloxacin, and ofloxacin are examples of quinolone antibiotics (Aldred et al., 2014).

Antibiotics such as macrolides, tetracyclines, and aminoglycosides can inhibit bacterial protein biosynthesis by interfering with different stages of translation (initiation, synthesis, and elongation phases) (Figure 1, a4). Macrolide antibiotics consist of a lactone ring with deoxy sugars (Li et al., 2022) and bind the peptidyl transferase center created by 23S ribosomal RNA (rRNA) at the 50S ribosomal subunit of bacteria, inhibiting the movement of the ribosome along the mRNA and thus preventing the elongation of the nascent polypeptide chain. This ultimately leads to the inhibition of bacterial protein synthesis and eventual bacterial cell death (Vazquez‐Laslop & Mankin, 2018). Common macrolide antibiotics include erythromycin, clarithromycin, and azithromycin (Monahan et al., 2023). Tetracycline antibiotics comprise a linear fused tetracyclic nucleus attached with different functional groups, bind to bacterial 30S ribosomal subunit, inhibit the binding of aminoacyl‐tRNA to the A site of the ribosome, and prevent the incorporation of amino acids into the growing polypeptide chain. Tetracycline, oxytetracycline, and doxycycline are the most used tetracycline antibiotics (Ye et al., 2021). Aminoglycoside antibiotics contain amino sugar substructures and have a strong affinity for the A‐site on the 16S rRNA of the 30S ribosome. Although mRNA binding and 50S subunit association occur, genetic code misreading at the mRNA leads to premature termination of the polypeptide chain, disrupting protein synthesis and causing cell death (Krause et al., 2016). Kanamycin, streptomycin, and gentamicin were one of the most frequently prescribed aminoglycoside antibiotics (Dillard et al., 2022).

Antibiotics interact with bacterial cell membranes to alter permeability. Lipopeptide antibiotics consist of a peptide core with a lipid tail. Experimental evidence suggests that lipopeptide antibiotics oligomerize and form micelles in the presence of calcium, which enables daptomycin molecules to embed into the bacterial cell membrane (Figure 1, a5) (Scott et al., 2007). Antibiotics with this action cause pore formation, depolarization, and cell death (Gutierrez‐Chavez et al., 2021). Presently, daptomycin stands as the sole approved lipopeptide antibiotic for clinical application (Ledger et al., 2022).

While the mechanisms by which antibiotics exert their antibacterial effects have been extensively studied, the ongoing development of new antibiotics is critical in addressing the growing threat of antibiotic resistance. In recent years, deep learning and artificial intelligence (AI) have facilitated the discovery of novel antibiotics by combining biological knowledge with computational models. For example, Swanson et al. (2024) introduced SyntheMol, a generative model that leverages Monte Carlo tree search algorithms to assemble novel compounds from extensive molecular building blocks. The model was used to create 58 molecules from a chemical space of nearly 30 billion molecules that inhibit *Acinetobacter baumannii*, with six of them showing antibacterial activity against multiple bacterial pathogens. These techniques overcome the limitations of traditional chemical screenings, allowing for the exploration of a broader chemical space and new structural motifs, thus opening new pathways for antibiotic development (Abbas et al., 2024). This emerging research not only enhances our understanding of antibiotic mechanisms but also helps to better understand the response of bacteria and the development of antibiotic resistance.

### 2.2 The mechanism of antibiotic resistance

Bacterial antibiotic resistance is a natural phenomenon as a result of microbial evolution over thousands of years. This inherent antibiotic resistance is also known as intrinsic resistance (Pawlowski et al., 2016). Apart from intrinsic resistance, the overuse of antibiotics can lead to the accumulation of antibiotic resistance (Razzaque, 2020). Bacteria employ various strategies to resist antibiotics, such as reducing antibiotic uptake, modifying or inactivating the antibiotics, actively expelling it from the cell, or altering the antibiotic's target to prevent its action (Figure 1) (Reygaert, 2018).

Bacteria attempt to restrict antibiotic access by changing or limiting entry to the cell. Gram‐negative bacteria have an outer lipid membrane, which can serve as a barrier to exclude antibiotics (β‐lactams, quinolones) from entering the cell (Breijyeh et al., 2020). Most antibiotics need to penetrate the outer lipid membrane to reach their targets. Hydrophobic drugs such as macrolides (erythromycin) can cross the membrane via diffusion, while hydrophilic antibiotics like β‐lactams need to go through porins (Figure 1, b1). Changes in the outer membrane, such as alterations to hydrophobic properties or mutations in porins, can lead to antibiotic resistance in Gram‐negative bacteria (Choi & Lee, 2019). Some bacteria can produce a substantial amount of extracellular polymeric substances, including exopolysaccharides, proteins, extracellular DNA, and lipids. These substances can form a physical barrier around bacterial cells within the biofilm, preventing antibiotics from directly reaching the bacterial cells (Abebe, 2020). This decreases direct antibiotic exposure, resulting in an apparent reduction of antibiotic efficacy. For example, the biofilms generated by *Staphylococcus aureus* strains could significantly diminish the penetration of oxacillin, vancomycin, and cefotaxime (Singh et al., 2010).

Bacteria can chemically modify or degrade antibiotics, rendering them ineffective, by producing enzymes that break down the antibiotic molecules (Darby et al., 2023). The most significant resistance mechanism against β‐lactams is the production of β‐lactamase enzymes by antibiotic‐resistant bacteria (Figure 1, b2). These enzymes inactivate β‐lactams by irreversibly opening the β‐lactams ring, which disables its ability to bind to penicillin‐binding proteins (Eiamphungporn et al., 2018). Likewise, enzymes like aminoglycoside acetyltransferase AAC(3) can chemically alter aminoglycoside antibiotics, such as gentamicin. This modification induces changes in the antibiotic structure, diminishing its ability to bind effectively to bacterial ribosomes and consequently reducing its efficacy (Serio et al., 2018).

Efflux pumps are transmembrane proteins able to remove antibiotics from the bacterial cell in an energy‐dependent manner and allow bacteria to survive in a high concentration of antibiotics. Bacterial drug efflux pumps can be classified into five families—ABC, SMR, MATE, MFS, and RND—based on the number of components, transmembrane‐spanning regions, energy source, and exported molecules (Figure 1, b3). Primary active transporters, such as ABC transporters, use energy obtained from adenosine triphosphate (ATP) breakdown, while secondary active transporters, including SMR, MATE, MFS, and RND, rely on the energy from the electrochemical gradient created by H^+^ (proton motive force) or Na^+^ (sodium motive force). Gram‐negative bacteria's intrinsic antibiotic resistance is primarily attributed to RND family efflux pumps. The increased expression of RND‐type efflux pumps, namely MexAB‐OprM, MexCD‐OprJ, MexEF‐OprN, and MexXY (‐OprA), has been documented as a significant factor contributing to decreased susceptibility of *Pseudomonas aeruginosa* to antibiotics, such as ticarcillin, ciprofloxacin, and meropenem (Zahedi Bialvaei et al., 2021). On the other hand, in Gram‐positive bacteria, the MFS family is the most prominent efflux pump group. For instance, MFS efflux pump NorA previously identified from *S. aureus* imparts resistance to chloramphenicol and fluoroquinolones (Sharma et al., 2019).

Changing the target binding site of the antibiotic can reduce the binding efficiency and lead to antibiotic resistance. 16S rRNA methyltransferases change the conformation of the ribosome's binding site for aminoglycosides and thus reduce the affinity of aminoglycosides for the ribosome, making it more difficult for antibiotics to bind effectively (Figure 1, b4) (McGann et al., 2016). Aminoglycoside‐modifying enzymes can be classified into two groups, methylate G1405 and A1408. The methylation of G1405 alters the structure of the ribosomal RNA at a critical binding site for these 4,6‐disubstituted 2‐deoxystreptamine (DOS) aminoglycosides (such as kanamycin, gentamicin, tobramycin, and amikacin). NpmA is the only acquired A1408 16S rRNA methyltransferase known to confer resistance to both 4,5‐ and 4,6‐disubstituted 2‐DOS aminoglycosides (Kawai et al., 2021).

Understanding the mechanisms behind antibiotic resistance has become increasingly sophisticated with advancements in molecular techniques. Among these, gene sequencing has emerged as a powerful tool, enabling the prediction of resistance phenotypes based on genetic data. This allows for more precise tracking of resistance evolution and informs targeted interventions to prevent the spread of resistant strains (Abbas et al., 2024). Additionally, bacteria employ a variety of strategies to ensure their survival, and even small numbers of resistant strains may persist in the complex microbial environment of the gastrointestinal tract, leading to the selection and survival of resistant bacterial populations (Isles et al., 2022). Understanding molecular resistance mechanisms is necessary to mitigate antibiotic resistance and unravel the dynamics of resistance accumulation in the human gut.

### 2.3 The spread and accumulation of antibiotic resistance in the gut

**FIGURE 2:**
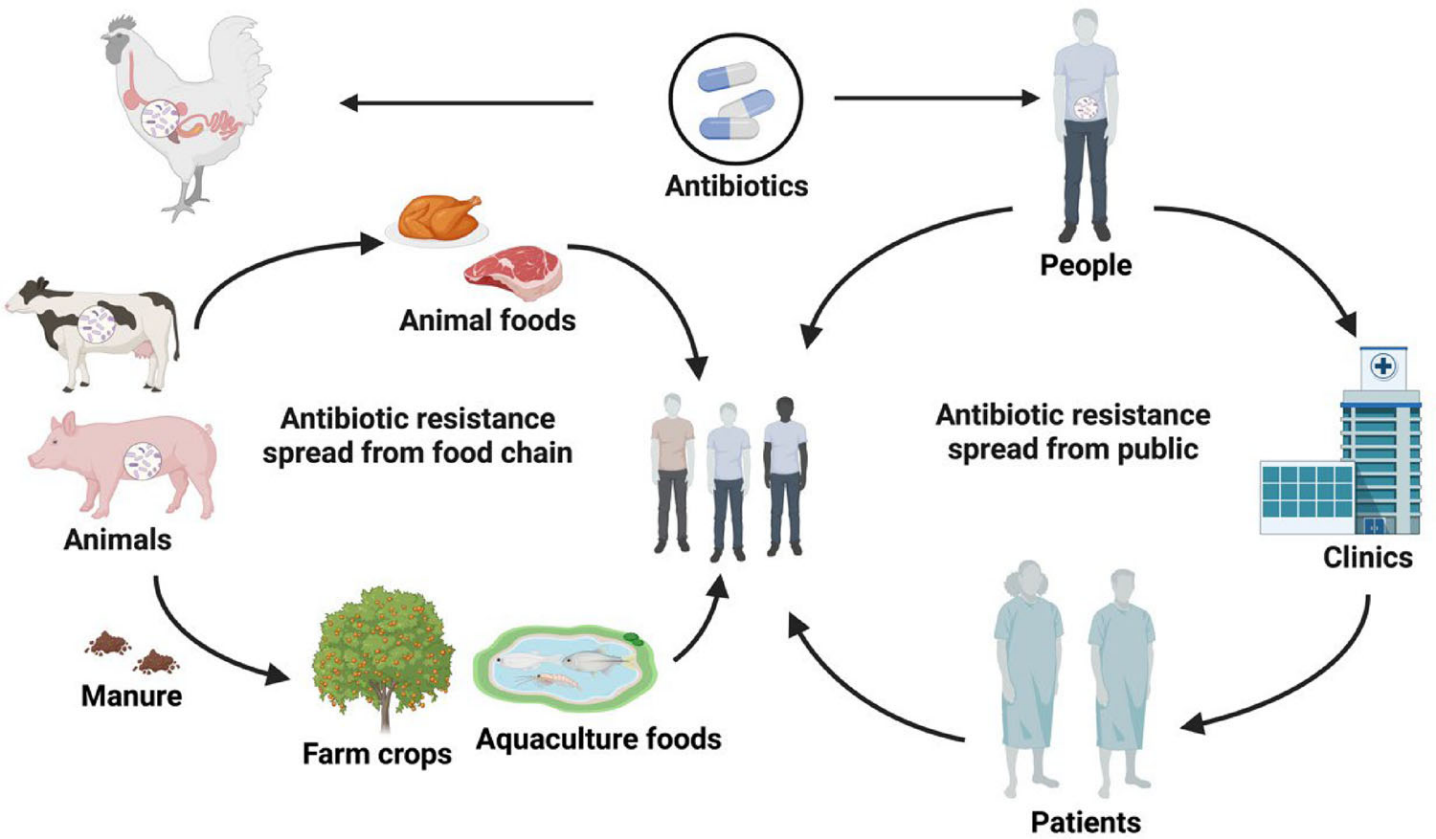
Accumulation of antibiotic resistance in the environment. Antibiotic resistance is present in natural settings like soil, water, and animal manure, as well as in community environments such as clinics. Antibiotic resistance transmits to humans via contaminated food and direct public contact.

Antibiotic resistance occurs in both natural environments (e.g., soils, water ecosystem, animal manure) and community environments (e.g., hospital effluents, agricultural practices) (Larsson & Flach, 2022). Antibiotic resistance can be transmitted from the natural environment to humans through the food chain when antibiotic‐resistant bacteria in foods are consumed by humans (Figure 2) (Kumar et al., 2020).

In the natural environment, there are connections between different organisms and substrates such as animals, soil, and water. This interconnection facilitates the movement of antibiotic resistance across various environmental components. Soil, water, and sand serve as reservoirs where resistant bacteria and genetic elements can persist and interact (Samreen et al., 2021). Moreover, the transmission of antibiotic resistance from the environment to plants, animals, and insects involves several mechanisms. Agricultural practices, including the use of antibiotics in animal husbandry and the application of manure from treated animals to fields, introduce resistance genes into the soil (Zhang et al., 2019). Water sources can become contaminated with antibiotic‐resistant bacteria and genetic elements through runoff from agricultural fields, particularly those fertilized with livestock manure (Zhu et al., 2023). Insects and other organisms in aquatic ecosystems can become carriers of resistance genes, further spreading antibiotic resistance within the environment (Rawat et al., 2023).

Human activities significantly contribute to the discharge of antibiotics into the environment. This occurs through multiple routes, including municipal and hospital waste, industrial manufacturing, agricultural runoff, and landfill leachates (Jampani et al., 2024). As a result, antibiotic resistance may arrive in the human gut through various pathways. Humans can be exposed to antibiotic‐resistant bacteria and genetic elements through contaminated food, water, and direct contact with the environment (e.g., familial, community, or healthcare interactions) (Brooks et al., 2023).

Given the widespread occurrence of antibiotic resistance in both natural and community environments, regulatory efforts have been implemented globally to mitigate its impact. These strategies focus on promoting the rational use of antibiotics, raising public awareness, improving antibiotic stewardship, and employing chemical methods to treat antibiotics and antibiotic‐containing waste (Barathe et al., 2024). Together, these approaches aim to reduce the spread of resistance and safeguard public health.

**FIGURE 3:**
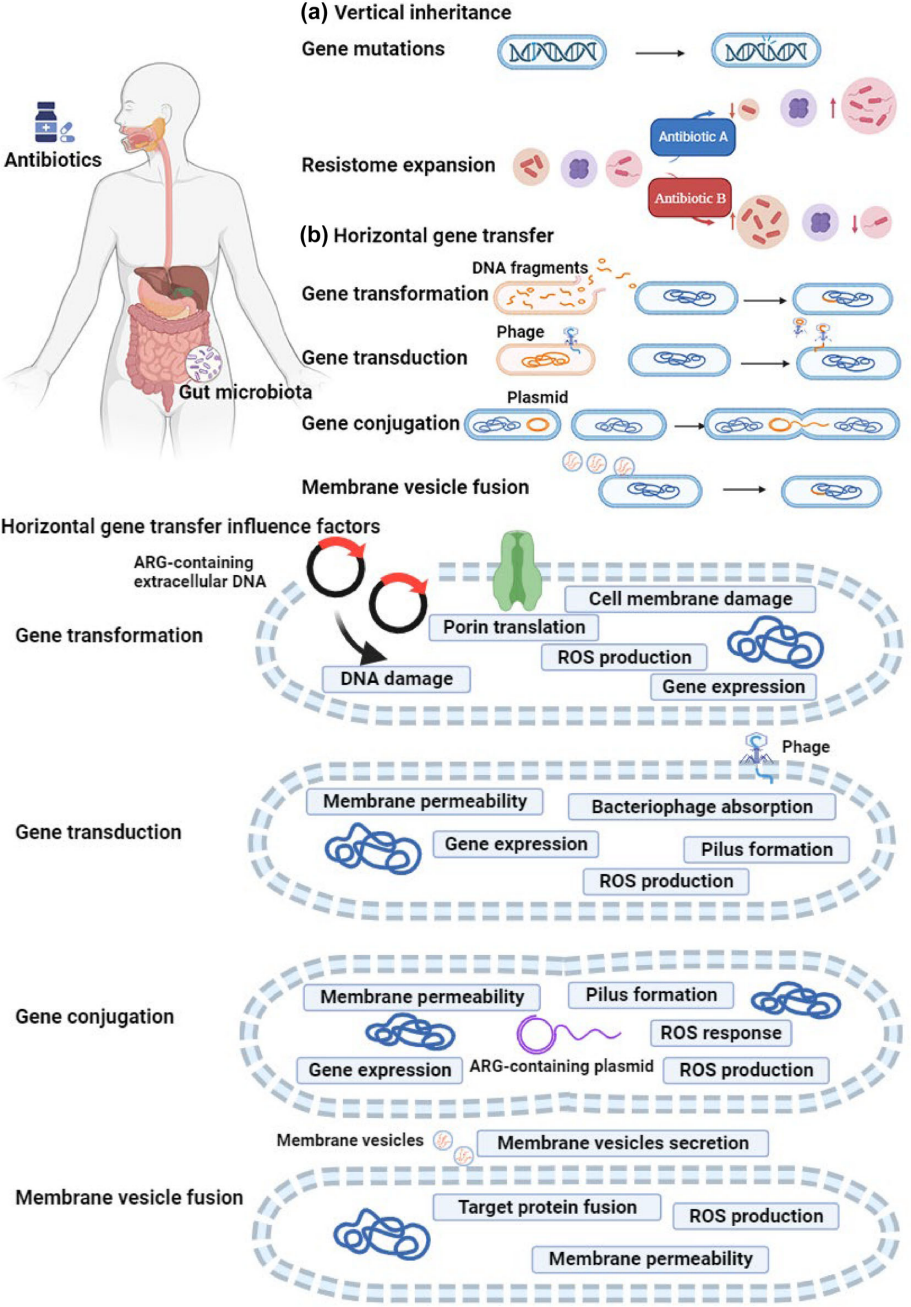
Accumulation of antibiotic resistance in the gut through vertical inheritance, horizontal gene transfer, and the factors that influence these mechanisms. (a) Vertical inheritance: Antibiotic resistance can be vertically inherited through gene mutation and resistome expansion. (b) Horizontal gene transfer and its influence factors: Horizontal gene transfer occurs in four pathways: Gene transformation, where free DNA fragments from a donor bacterium are taken up by a recipient bacterium, influenced by factors such as porin translation, cell membrane damage, gene expression, DNA damage, and reactive oxygen species (ROS) production; gene transduction, involving the transfer of DNA via a temperate phage, affected by pilus formation, bacteriophage absorption, membrane permeability, gene expression, and ROS production; gene conjugation, where genetic material is transferred through pili connecting donor and recipient bacteria, influenced by pilus formation, membrane permeability, SOS response, gene expression, and ROS production; and membrane vesicle fusion, where vesicles from the donor cell transport antibiotic resistance genes (ARGs) into the recipient cell's cytoplasm by fusing with its membrane, driven by membrane vesicle secretion, membrane permeability, target protein fusion, and ROS production.

The occurrence, accumulation, and spread of ARGs in the natural environment, animals, and humans rely on vertical inheritance and horizontal gene transfer (Figure 3) (Sommer et al., 2017). During the growth and reproduction of bacteria, genetic mutations allow them to obtain resistance to specific antibiotics, and their offspring will inherit the mutated gene (Figure 3a) (Roberts et al., 2021). Bacteria carrying the mutated genes, considered ARGs, are more likely to survive and can be spread vertically from generation to generation. Over time, the population of bacteria will become dominated by those with the resistance gene, and this selective advantage allows resistant bacteria to proliferate, contributing to the expansion of the antibiotic resistome (Kim & Cha, 2021).

ARGs have high mobility and can be spread through horizontal gene transfer, which includes four mechanisms: gene transformation, gene transduction, gene conjugation, and membrane vesicle fusion (Figure 3b) (Tao et al., 2022). Antibiotics can influence horizontal gene transfer in bacteria, particularly in the context of ARGs. They create a selective environment favoring the survival and proliferation of bacteria carrying resistance genes (Li & Zhang, 2022). Apart from antibiotic selective pressure, various nonantibiotic factors, including antimicrobial agents and environmental conditions, can influence the horizontal transfer of ARGs (Jiang et al., 2022).

Gene transformation refers to the direct uptake of free DNA fragments of ARGs from a donor bacterium by a recipient bacterium, so that the recipient bacteria can acquire antibiotic resistance. Wang et al. (2020) discovered that nonantibiotic pharmaceuticals, including ibuprofen, naproxen, diclofenac, gemfibrozil, and propranolol, can markedly increase the frequency of horizontal gene transfer of ARGs by gene transformation. This phenomenon is attributed to the ability of nonantibiotic pharmaceuticals to elevate stress levels, trigger the overproduction of reactive oxygen species (ROS), enhance cell membrane permeability, and enable the cells to incorporate exogenous DNA, including ARGs.

Gene transduction refers to the transfer of DNA from a donor bacterium into a recipient bacterium using a temperate phage as a carrier. For gene transduction, it has been observed that when nano‐TiO_2_ undergoes photoexcitation, damage to the cell membrane caused by extracellular ROS and the induction of pilus synthesis by intracellular ROS both play roles in contributing to phage invasion. Additionally, these processes facilitate the horizontal transfer of ARGs through phage transduction (Xiao et al., 2021).

Gene conjugation refers to the transfer of genetic material (such as plasmid DNA) from a donor bacterium to a recipient bacterium by connecting bacteria through pili. Zhang et al. (2023) discovered that fungicides have the potential to act as selectors for the spread of ARGs through plasmid‐mediated conjugation. Specifically, exposure to chlorothalonil mainly triggered the generation of intracellular ROS, stimulated the ROS response, and heightened cell membrane permeability. In contrast, azoxystrobin and carbendazim primarily boosted the expression of flagellin genes, increasing bacteria mobility and contact between individual cells and thereby promoting plasmid conjugation transfer.

Membrane vesicle fusion is characterized by the secretion of membrane vesicles from the donor cell's surface, which then transport DNA containing ARGs into the recipient cell's cytoplasm through fusion with its cytomembrane. During the fusion process, the membrane of these vesicles combines with the membrane of the target cell, leading to the delivery of DNA into the recipient cell. This fusion event not only facilitates DNA transfer but also promotes the horizontal transfer of ARGs. The particle sizes and concentrations of vesicles, as well as their molecular interactions, influence DNA packaging and delivery, as evidenced by the fact that larger size and higher concentration of vancomycin‐resistant enterococci could release significantly more vesicles under vancomycin stress (Lehmkuhl et al., 2024).

In most bacteria, the dominant and most efficient method of acquiring resistance genes involves the use of mobile genetic elements such as plasmids as vectors for gene exchange (Vinayamohan et al., 2022). Horizontal gene transfer has been observed in a variety of bacteria, such as *Escherichia coli* and *Klebsiella pneumoniae*, allowing resistance genes to spread rapidly and efficiently throughout bacterial populations (Frazao et al., 2019; Pedersen et al., 2020). In general, horizontal transfer of ARGs can exacerbate the spread of antibiotic resistance in the gut.

Overall, vertical inheritance and horizontal gene transfer are the two main pathways leading to the occurrence and spread of ARGs in the natural environment, farm animals, and eventually the human gut. Notably, horizontal gene transfer of ARGs is the key factor determining its spread. Food intervention plays a role in inhibiting horizontal gene transfer of ARGs. It has been found that some dietary phytochemicals such as phenolic compounds, sulfides, terpenoids, and alkaloids have the potential to induce the production of ROS, resulting in irreversible damage to the bacterial cell membrane, diminished biofilm viability, and downregulation of virulence gene expression, thereby inhibiting horizontal gene transfer (Goncalves et al., 2023). The role of food components in controlling ARGs will be further discussed in Section 4.

## 3 INFLUENCE OF GUT RESISTOME ON HUMAN HEALTH

When treating diseases with antibiotics, the method of administration significantly impacts the gut microbiome and resistome. Antibiotics administered intravenously (injected into the bloodstream) or topically (applied directly to the skin) generally have minimal direct contact with the gut microbiome and thus exert less influence on the gut resistome. However, oral antibiotics, which are taken by mouth, the most common form of treatment, pass through the digestive system, directly affecting the gut microbiota. This interaction can disrupt the balance of gut microorganisms (dysbiosis) and promote the emergence of ARGs (Kelly et al., 2020).

Zhang et al. (2013) highlighted this difference, showing that oral administration of tetracycline or amoxicillin in mice rapidly increased the presence of ARGs in the gut, while intravenous administration delayed or reduced this effect. This discrepancy is linked to the drugs’ excretion routes: ampicillin, excreted mainly through the kidneys, limits gut exposure after intravenous use, while tetracycline, eliminated via both the kidneys and bile, exposes the gut even after intravenous administration. These findings underscore the significant role of oral antibiotics in shaping the gut resistome.

**FIGURE 4:**
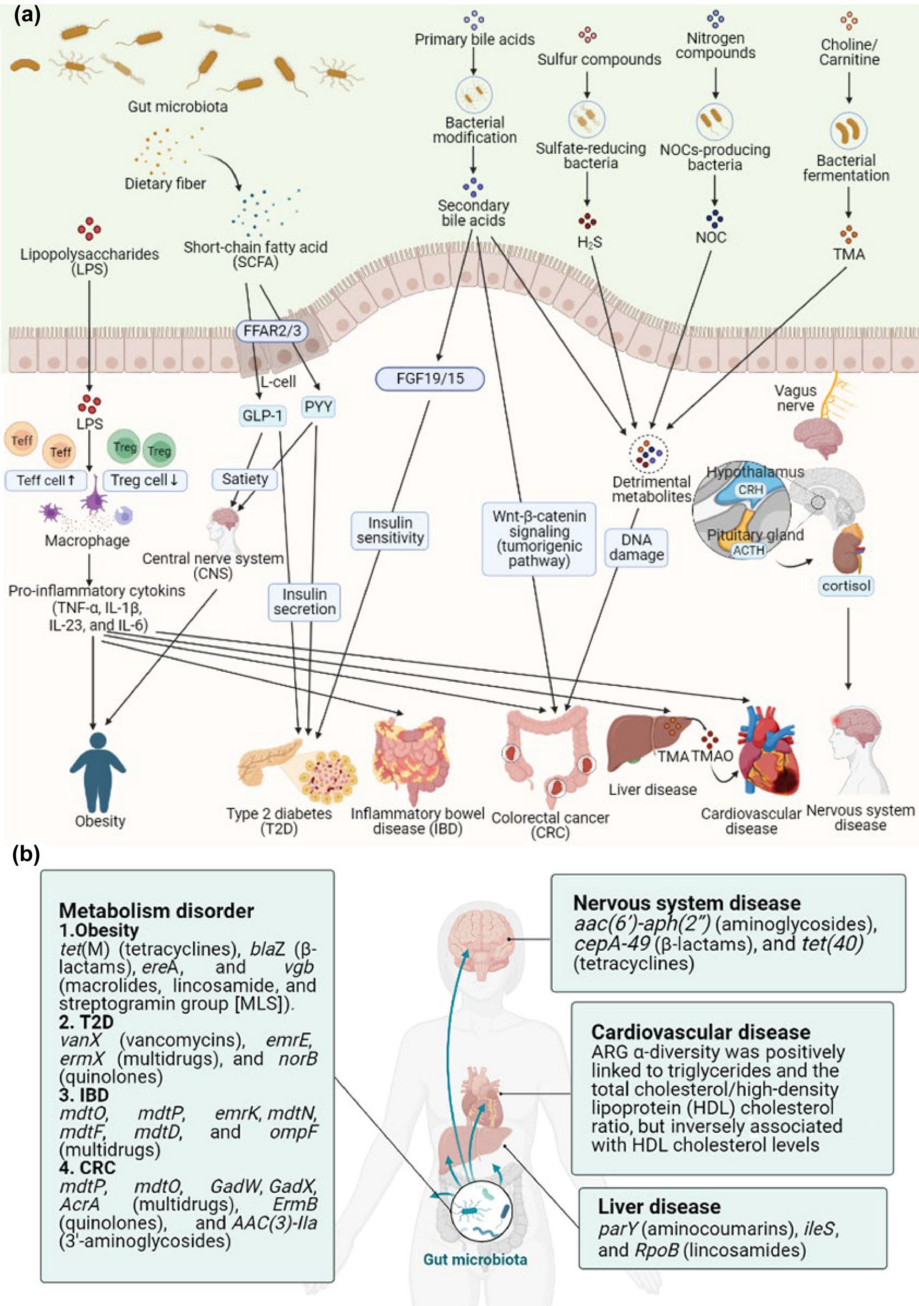
Influence of gut resistome on human health. (a) The mechanism of gut microbiota in health and disease. Short‐chain fatty acids (SCFAs) derived from dietary free fatty acid receptors (FFAR2/3) to promote satiety by releasing gut hormones like peptide YY (PYY) and glucagon‐like peptide 1 (GLP‐1) while enhancing insulin secretion and sensitivity, leading to obesity and type 2 diabetes (T2D). Lipopolysaccharides (LPSs) from Gram‐negative bacteria trigger systemic inflammation by activating macrophages and the release of pro‐inflammatory cytokines (tumor necrosis factor‐alpha [TNF‐α], interleukin‐1 beta [IL‐1β], and IL‐6), leading to obesity, T2D, inflammatory bowel disease (IBD), colorectal cancer (CRC), cardiovascular disease, and liver disease. Secondary bile acids, trimethylamine‐*N*‐oxide (TMAO), hydrogen sulfide (H_2_S), and nitrogen‐containing organic compounds (NOCs) contribute to dysregulation of bile acid signaling, oxidative stress, and DNA damage, leading to chronic diseases like CRC. Additionally, interactions between the gut and central nervous system (via the vagus nerve) and gut‐derived metabolites (such as trimethylamine (TMA) and TMAO) affect neurological and cardiovascular health. (b) The influence of antibiotic‐resistant genes on diseases. Specific antibiotic resistance genes (ARGs) are associated with metabolism disorder, nervous system disease, and liver disease. ARG α‐diversity is linked to increased cardiometabolic risks.

Moreover, extensive use of antibiotics either directly in humans or indirectly in animals induces the accumulation of antibiotic resistance in the natural environment and living creatures, thus contributing to the expansion of human gut resistome. Severe health problems have been linked to gut resistome, including metabolism disorder, cardiovascular disease, liver disease, and nervous system disease (Figure 4).

### 3.1 Metabolism disorders

The antibiotic resistome plays a crucial role in metabolic disorders, including obesity, type 2 diabetes (T2D), inflammatory bowel disease (IBD), and colorectal cancer (CRC), by disrupting gut microbiota and its associated metabolic pathways. The gut resistome, being part of the gut microbiome, has also been implicated in the development of these disorders.

In obese people, gut dysbiosis affects energy metabolism and chronic inflammation through alteration in SCFAs and lipopolysaccharides (LPSs) (Figure 4a) (Wang & He, 2018). SCFAs regulate satiety by stimulating the release of hormones like GLP‐1 and PYY, which act on the hypothalamus (Aoun et al., 2020). Moreover, an imbalance in gut bacteria, particularly an increased abundance of Gram‐negative bacteria such as *Proteobacteria*, leads to elevated LPS levels and triggers systemic inflammation by increasing the production of pro‐inflammatory cytokines (tumor necrosis factor‐alpha [TNF‐α], interleukin‐1 beta [IL‐1β], and IL‐6) (Shuai et al., 2022). In a study conducted by Sarmiento et al. (2019), a higher abundance of *Fusobacterium*, *Enterococcus*, and *E. coli* was observed in obese individuals compared with eutrophic participants, alongside elevated levels of ARGs, such as *tet*(M) (tetracyclines), *bla*Z (β‐lactams), *ere*A, and *vgb* (macrolides, lincosamide, and streptogramin group [MLS]) (Figure 4b). This suggests a relationship between ARGs and Gram‐negative bacteria driven by an imbalance between *Bacteroidetes* and *Proteobacteria*.

In T2D, gut microbial dysbiosis, including changes in the resistome, resulted in reduced SCFA production, impairing PYY and GLP‐1 secretion, which are vital for insulin regulation and glucagon levels (Figure 4a) (Zhang, Chu, et al., 2021). Additionally, alternations in bile acid metabolism stimulate the release of fibroblast growth factor 19/15 (FGF19/15), further contributing to insulin sensitivity and glucose tolerance (Zhang, Chu, et al., 2021). Similarly, Shuai et al. (2022) found that the increased ARG richness, including *Vancomycin_vanX*, *Multidrug_emrE*, *MLS_ermX*, and *Quinolone_norB*, was correlated with a higher risk of T2D, with changes in the resistome occurring before the alternations in the gut microbiota (Figure 4b).

In IBD, reduced SCFA‐producing bacteria impair the anti‐inflammatory function of SCFAs, which normally support the differentiation of regulatory T‐cells (Treg) and effector T‐cells (Teff) (Figure 4a) (Lee & Chang, 2021). This reduction promotes inflammation and epithelial damage, creating an environment that favors pathogenic bacteria and perpetuates chronic inflammation (Zhang et al., 2022). Xia et al. (2021) reported a higher prevalence of ARGs, such as *mdtO*, *mdtP*, *emrK*, *mdtN*, *mdtF*, *mdtD*, and *ompF*, carried by *E. coli*, which contribute to increased gut permeability and promote inflammation by impairing the intestinal barrier (Figure 4b). ARGs and virulence factors spread within the microbiome through horizontal gene transfer, exacerbating inflammation in IBD.

In CRC, gut dysbiosis contributes to cancer initiation and progression by reducing mucus layer thickness and increasing intestinal permeability (Figure 4a). Overgrowth of harmful bacteria leads to the release of LPSs and other toxic metabolites like secondary bile acids, trimethylamine‐*N*‐oxide (TMAO), hydrogen sulfide (H_2_S), and nitrogen‐containing organic compounds, which trigger low‐grade inflammation and activate tumorigenic signals (Zhang, An, et al., 2021). Yuan et al. (2021) observed elevated levels of *Escherichia*, *Citrobacter*, and *Acinetobacter* in CRC patients, along with resistant genes like *mdtP*, *mdtO*, *GadW*, *GadX*, *AcrA* (multidrug) *ErmB* (quinolone), and *AAC(3)‐IIa* (3′‐aminoglycoside) (Figure 4b). These ARGs contribute to CRC progression by promoting dysbiosis, inflammation, and gut barrier dysfunction, highlighting the role of gut resistome in the pathogenesis of metabolic disease.

Overall, the emerging evidence suggests that the gut resistome not only reflects microbial imbalance but also actively drives metabolic disorders through the propagation of antibiotic resistance. The presence and spread of ARGs within the gut microbiota disrupt key physiological processes, such as energy metabolism, immune modulation, and gut barrier integrity. By fostering an environment conducive to pathogenic bacteria and their virulence factors, the resistome amplifies inflammation, alters metabolic signaling pathways, and promotes dysbiosis.

### 3.2 Cardiovascular disease

The gut microbiome contributes to the pathogenesis of cardiovascular disease, including heart failure and coronary artery disease, through inflammation reactions and TMAO metabolism mechanism (Figure 4a). Patients with cardiovascular disease have been shown to exhibit a decreased abundance of gut microbes capable of producing butyrate, which promote local inflammation, exacerbate dysbiosis, and contribute to impaired gut barrier function (Jie et al., 2017). Elevated TMAO levels in these patients contribute to the development of atherosclerosis by interfering with cholesterol transportation, promoting foam cell formation, and inducing platelet aggregation, potentially leading to acute coronary syndromes (Troseid et al., 2020).

Additionally, higher gut ARG α‐diversity is linked to increased cardiometabolic risks, as Shuai et al. (2022) found that higher gut ARG α‐diversity indices were positively associated with elevated triglycerides and the total cholesterol/high‐density lipoprotein (HDL) cholesterol ratio but inversely associated with HDL cholesterol levels. This highlights the role of ARGs in disrupting lipid and glucose metabolism and increasing the likelihood of cardiovascular disease through dyslipidemia and insulin resistance.

### 3.3 Liver disease

The gut microbiota exerts a significant influence on liver physiology by inflammation reactions (Figure 4a). Alternations in the gut microbiome, influenced by the antibiotic resistome, can increase intestinal permeability, allowing bacteria and their byproducts, including LPSs, to enter the liver via hepatoenteric circulation. LPS exposure triggers acute inflammatory responses, leading to liver damage and contributing to diseases like nonalcoholic fatty liver disease, steatohepatitis, alcoholic liver disease, hepatocarcinogenesis, and hepatic encephalopathy (HE) (Ahlawat et al., 2021).

Changes in the gut resistome are also closely associated with liver disease. Shamsaddini et al. (2021) found patients with cirrhosis exhibited a higher abundance of resistome associated with pathobionts such as Enterobacteriaceae, *Streptococcus*, *Enterococcus*, and *Acinetobacter* spp. Additionally, they showed greater resistance patterns related to β‐lactamases, macrolides, quinolones, glycopeptides, fosfomycin, and tetracyclines. Patients with HE also exhibited higher levels of aminocoumarin‐resistant *parY* and lincosamide resistance (*ileS* and *RpoB*). These resistome changes contribute to disruptions in the gut–liver axis and gastrointestinal immune response and worsen with disease progression.

### 3.4 Nervous system disease

The gut microbiome plays a pivotal role in shaping the development and function of the nervous system through the gut–brain axis. The vagus nerve transmits neuronal, endocrine, and immune signals, while the hypothalamus regulates stress responses via the release of corticotropin‐releasing hormone (CRH), leading to cortisol production, which affects the gut's epithelial barrier and immune system (Mitrea et al., 2022).

Alterations in the gut resistome have also been linked to neurodevelopmental disorders. Kovtun et al. (2020) found children with autism spectrum disorder (ASD) had a higher abundance of the *aac(6′)‐aph(2′’)* gene from *Enterococcus faecium*, *cepA‐49* and *tet(40)* gene from *Megasphaera elsdenii*, and *cepA‐49* gene from *Bacteroides fragilis*. The study found statistically significant alterations between healthy individuals and those with ASD in genes conferring resistance to macrolides from *Bacteroides*, *Campylobacter*, and *Clostridium*; aminoglycoside resistance genes from *Enterococcus* and uncultivated bacteria; β‐lactam resistance genes from *Bacteroides* and *Acidaminococcus*; and tetracycline resistance genes from *Megasphaera* and *Alistipes*. Therefore, disruption of the gut microbiota balance and the resistant genes could diminish neurometabolic potential, impacting the production of essential neurotransmitters and metabolites that influence brain function.

Diseases commonly treated with antibiotics are associated with expanded gut resistomes, suggesting that historical exposure to antibiotics has exerted considerable selective pressure for ARG acquisition in disease‐associated strains (Fredriksen et al., 2023). Although gut microbiota has been well associated with various diseases, such as metabolism disorder, cardiovascular disease, liver disease, and nervous system disease, there is limited research targeting the effects of ARGs on the progression of human diseases and the influence of disease on the accumulation of ARGs in the gut. Future research is required to better understand the interaction between gut resistome and diseases, as well as the mechanism of action. Looking ahead, integrating polygenic risk scores with gut metagenomic risk models could improve the predictive power for common chronic diseases, offering a more holistic approach to disease prediction and prevention (Liu et al., 2024).

## 4 ALTERATION OF GUT RESISTOME THROUGH DIETARY COMPONENTS

The alteration of the gut resistome through dietary components is an emerging area of research, drawing increasing attention to how food choices influence antibiotic‐resistant bacteria in the gut. Fermented foods, for instance, contain significant populations of microorganisms, with lactic acid bacteria and coagulase‐negative *Staphylococcus* identified as carriers of ARGs against tetracyclines, penicillins, chloramphenicol, and macrolides (Pop et al., 2024). This suggests that the consumption of fermented foods may directly impact the gut resistome.

Many natural molecules present in food such as phenolics, terpenes, and phenolic amides have been demonstrated with antimicrobial ability and are able to regulate gut microbiota (Jiang et al., 2022; Leonard et al., 2022, 2021; Liang et al., 2023). Nevertheless, the research on the impacts of food components on antibiotic‐resistant bacteria in the gut is limited, representing an emerging research area. This article summarizes nutritional studies on a large population to understand the potential influences of dietary habits on gut resistome and highlights the modulatory effect of selected categories of dietary components on antibiotic‐resistant bacteria.

### 4.1 Long‐term diet habit may alter gut resistome

Long‐term dietary habits have been found to influence both gut microbiota and gut resistome. Stege et al. (2022) selected 149 Dutch individuals with dietary habits of four distinct groups—omnivores, pescatarians, vegetarians, and vegans—and found that long‐term dietary habits did not significantly influence the key composition and diversity of the gut microbiome. No significant differences were observed in the top 10 most abundant ARGs. Despite this, two ARGs (*lsa(C)* and *tet(L)*) showed differential abundances between omnivores and pescatarians. Additionally, metagenomic analysis revealed that *tet(X)* was more abundant in the resistome of omnivores compared to pescatarians. Similar results were observed among 58 volunteers from three different eating habits (omnivorism, ovolactovegetarianism, and strict vegetarianism) in their gut resistome (da Silva et al., 2021). The most prevalent resistance genes among all habit groups were against tetracyclines (*tet(A)*, *tet(B)*, *tet(M)*, *tet(O)*, *tet(Q)*), β‐lactams (*blaTEM*, *blaSHV*, *mef*), and the MLS resistance genes (*erm(B)*, *erm(C)*), followed by sulfonamide (*sul1*, *sul2*) and aminoglycoside resistance genes (*aacA‐aphD*). *blaCTX‐M* was exclusively found in samples from the ovolactovegetarian group, while *tet(E)* was only detected in samples from the omnivore group. Interestingly, these frequently detected ARGs were also commonly found in farm animal feces, indicating the potential linkage of diet habits and environment ARGs.

While the exact mechanism linking diet to gut resistome remains unknown, it is plausible that dietary interventions could offer a promising approach to alleviate the burden of antibiotic resistance. Oliver et al. (2022) studied 290 healthy adults to explore the relationship between diet, gut microbiome, and antibiotic resistance and observed lower levels of ARGs in individuals with high dietary fiber intake. The low‐ARG population group showed more obligate anaerobes from the *Clostridiaceae* family in their gut, while the high‐ARG group had higher levels of *Streptococcaceae* and *Enterobacteriaceae*. Moreover, low‐ARG individuals consumed less protein, especially from beef and pork sources. The study suggested that increasing fiber intake might promote a gut microbiome environment that favors the growth of obligate anaerobes while limiting facultative anaerobes, thus potentially reducing the prevalence of antibiotic resistance in the gut. This occurs because dietary fibers fermented by obligate anaerobes produce SCFAs that lower the pH of the gut environment, making it favorable for obligate anaerobes but inhospitable for facultative anaerobes.

### 4.2 Dietary components can inhibit antibiotic‐resistant bacteria

Specific dietary components play a crucial role in regulating gut microbiota through various mechanisms. Bioactive macronutrients like lectins, lactoferrin, and polyunsaturated fatty acids (PUFAs), derived from carbohydrates, proteins, and lipids, are primarily recognized for their nutritional roles, but they also possess bioactive properties that inhibit antibiotic bacteria by disrupting nutrient availability and bacterial membrane integrity (Kussmann et al., 2023). Phytochemicals like polyphenols exhibit antimicrobial properties by disrupting cellular functions, interfering with metabolic processes, or inhibiting enzyme activity (Yadav et al., 2023). Probiotics, such as *Lactobacillus* and *Bifidobacterium*, inhibit pathogens through competitive exclusion, secretion of antimicrobial compounds, and immune modulation (Maukonen & Saarela, 2014).

Interestingly, some of these compounds also possess the ability to selectively inhibit antibiotic‐resistant bacteria by targeting their vulnerabilities, such as impairing biofilm formation, inhibiting resistance‐conferring enzymes, and disrupting efflux pumps (Padilha da Silva et al., 2024). Investigating how these compounds specifically target bacteria, including antibiotic‐resistant strains, is vital for developing dietary strategies that control or reduce pathogenic populations in the gut. This research is significant as it offers a nonantibiotic approach to managing gut health and helps curb the spread of antibiotic resistance.

#### 4.2.1 Bioactive macronutrients

Macronutrients, which include carbohydrates, proteins, and fats, are essential components of the human diet that support life and regulate various physiological functions. While their primary role is to provide energy and building blocks for the body, certain bioactive macronutrients also have inherent antibacterial properties. These properties may help control the growth of antibiotic‐resistant bacteria and mitigate their impact on the gut microbiome, potentially offering an alternative strategy to combat antibiotic resistance (Table 1).

**TABLE 1:** Influence of food on antibiotic‐resistant bacteria.

Category	Food component	Target resistant bacteria	Inhibitory mechanism	Reference
Macronutrients				
	Carbohydrates (lectin)	Multidrug‐resistant *Staphylococcus aureus* and *Pseudomonas aeruginosa*	Increase antibiotic bioavailability	Santos et al., 2023
	Protein (lactoferrin)	Multidrug‐resistant *Acinetobacter baumannii*	Inhibit biofilm formation	Avery et al., 2021
	Protein (antimicrobial peptides)	Methicillin‐resistant *Staphylococcus aureus*	Disrupt bacterial membranes	Heymich et al., 2021
	Dietary fats (arachidonic acid, docosahexaenoic acid)	Multidrug‐resistant *Acinetobacter baumannii*	Enhance membrane fluidity and increase antibiotic bioavailability	Zang et al., 2021
Phytochemicals				
	Polyphenols (persimmon tannin)	Methicillin‐resistant *Staphylococcus aureus*	Reduce membrane potential and disrupt protein integrity	Liu et al., 2019
	Polyphenols (epigallocatechin gallate)	Multidrug‐resistant *Staphylococcus aureus* and *Escherichia coli*	Disrupt bacterial cell membranes	Parvez et al., 2019
	Polyphenols (proanthocyanidins)	Methicillin‐resistant *Staphylococci*	Increase antibiotic bioavailability	Gallique et al., 2021
	Sulfur‐based compounds (allicin)	Multidrug‐resistant *Staphylococcus aureus*	Inhibit biofilm formation	Xu et al., 2018
	Sulfur‐based compounds (isothiocyanates)	Multidrug‐resistant *Pseudomonas aeruginosa*	Inhibit biofilm formation	Kaiser et al., 2017
	Sulfur‐based compounds (saponins)	Multidrug‐resistant *Pseudomonas aeruginosa* and *Staphylococcus aureus*	Alter membrane permeability	da Silva et al., 2024
	Terpenoids (1, 8‐cineol)	Carbapenem‐resistant *Klebsiella pneumoniae*	Enhance outer membrane permeability and induce oxidative stress	Moo et al., 2021
	Terpenoids (linalyl anthranilate)	Carbapenem‐resistant *Klebsiella pneumoniae*	Induce oxidative stress and damage bacterial membrane	Yang et al., 2021
	Terpenoids (carnosic acid, carnosol, and 12‐methoxy‐*trans*‐carnosic acid)	Multidrug‐resistant *Staphylococcus aureus*	Inhibit efflux pump activity	Oluwatuyi et al., 2004
	Alkaloids (protoberberine alkaloids)	Multidrug‐resistant *Escherichia coli*, *Enterobacter aerogenes*, *Klebsiella pneumoniae*, *Providencia stuartii*, and *Staphylococcus aureus*	Inhibit the H+‐ATPase pump and decrease cellular ATP production	Guefack et al., 2022
	Alkaloids (matrine)	Multidrug‐resistant *Pseudomonas aeruginosa*	Inhibit biofilm formation and efflux pump activity	Pourahmad Jaktaji & Koochaki, 2022
	Alkaloids (6‐(2′‐methylbutyryloxy)‐3‐hydroxytropane and 6‐butyryloxy‐3‐hydroxytropane)	Multidrug‐resistant *Staphylococcus aureus*	Impair ABC multidrug transporters	Barbosa et al., 2021
Probiotics				
	Yogurt (*Lactobacillus parafarraginis*)	Multidrug‐resistant *Escherichia coli, Pseudomonas aeruginosa, Acinetobacter baumannii*, *Enterobacter aerogenes*, *Proteus mirabilis*, and *Klebsiella pneumoniae*	Produce bacteriocin‐like peptides	Gomez‐Gallego et al., 2018
	Kimchi (*Pediococcus inopinatus* K35)	Multidrug‐resistant *Pseudomonas aeruginosa*	Inhibit biofilm formation	Yi & Kim, 2023

Carbohydrates are not only a source of energy but also possess bioactive properties that can enhance the effectiveness of antibiotics. Mannose‐binding lectin from *Dioclea violacea* seeds has been shown to enhance antibiotic efficacy. Santos et al. (2023) demonstrated that lectin extracted from *D. violacea* enhanced the activity of neomycin against multidrug‐resistant *S. aureus* and *P. aeruginosa* by acting as an adjuvant, increasing the bioavailability of aminoglycosides near the bacterial membrane through carbohydrate recognition, and facilitating the entry of the antibiotic into the bacterial cytoplasm.

Proteins, such as lactoferrin found in human and bovine milk, also exhibit antibacterial and antibiofilm properties. Avery et al. (2021) reported that both human and bovine lactoferrin could substantially suppress the formation of biofilm in tested multidrug‐resistant *A. baumannii* strains, while human lactoferrin exhibited a lower minimum inhibitory concentration (MIC) than bovine lactoferrin, indicating a higher potency. In addition to lactoferrin, antimicrobial peptides (AMPs) derived from plants have shown promise in combating antibiotic‐resistant bacteria. Heymich et al. (2021) identified 21 AMP candidates from chickpea, two of which exhibited bactericidal activity against methicillin‐resistant *S. aureus* ATCC 3300. These peptides interact with bacterial membranes, leading to cell lysis through membrane disruption, which is directly correlated with their bactericidal effects.

Dietary fats, particularly PUDAs, can also improve the effectiveness of certain antibiotics. Zang et al. (2021) found that PUFAs, such as arachidonic acid (AA) and docosahexaenoic acid (DHA), enhanced the effectiveness of aminoglycosides against multidrug‐resistant *A. baumannii*. Both AA and DHA mitigated the antimicrobial effects of colistin by extracellular sequestration, making it hard to penetrate the bacterium's hydrophilic capsule and lipooligosaccharide barriers, and upregulated efflux system gene expression (*adeABC* and *adeIJK*), which increased bacterial resistance by expelling antibiotics from the cell.

The above studies highlight the role of bioactive macronutrients in both enhancing the effectiveness of antibiotics and influencing resistance mechanisms. Carbohydrates like pectin can improve antibiotic efficacy by interacting with antibiotics, while proteins like lactoferrin exhibit potent antibacterial and antibiofilm properties. However, dietary fats like AA and DHA present a more nuanced role, as they can both enhance antibiotic action and upregulate bacterial resistance mechanisms through the activation of efflux pumps.

In conclusion, bioactive macronutrients do not merely serve as energy sources but may also offer significant potential in the fight against antibiotic‐resistant bacteria. While their roles in modulating bacterial resistance mechanisms are complex, these findings underscore the importance of further research into the potential of dietary components to complement or enhance traditional antibiotic therapies. By leveraging these natural compounds, we may be able to develop novel strategies to mitigate the growing challenge of antibiotic resistance.

#### 4.2.2 Phytochemicals

Phytochemicals originate from plant‐based foods and mainly include polyphenols, sulfur‐containing compounds, terpenoids, and alkaloids (Pawase et al., 2024). These compounds have been found to display antimicrobial activity against antibiotic‐resistant bacteria, with some disrupting bacterial membrane structures and others enhancing antibiotic effectiveness by inhibiting efflux pumps, thereby preventing the development of resistance (Table 1) (Khameneh et al., 2021).

One well‐studied polyphenol, persimmon tannin from young astringent persimmon fruit, has shown inhibitory effects against methicillin‐resistant *S. aureus* (Liu et al., 2019). Persimmon tannin suppressed cell proliferation, decreased membrane potential and intracellular ATP concentration, disrupted whole‐cell protein integrity, and induced S‐phase cell cycle arrest in cells. In addition to their direct antimicrobial effects, some polyphenols can enhance the efficacy of antibiotics. Parvez et al. (2019) reported a pronounced synergistic effect when epigallocatechin gallate (EGCG) was combined with gentamicin, enhancing antibiotic efficacy against multidrug‐resistant strains of *S. aureus* and *E. coli*. This is because EGCG could disrupt bacterial cell membranes, hinder DNA supercoiling, increase cell permeability, ultimately culminate in bacterial demise, and mitigate the development of resistance. Similarly, proanthocyanidins extracted from American cranberry (*Vaccinium macrocarpon* L.) have shown significant synergy with β‐lactam antibiotics. Gallique et al. (2021) reported that proanthocyanidins inhibited β‐lactamases and selectively enhanced the effectiveness of oxacillin and carbenicillin against methicillin‐resistant *Staphylococci*, possibly via the modulation of the variant transpeptidase PBP2a expression in *Staphylococcus* species. Lower expression of PBP2a diminishes the bacteria's ability to resist β‐lactam antibiotics, making bacteria more susceptible to the antibiotics.

Sulfur‐based phytochemicals are also renowned for their ability to disrupt pathogenic biofilms and inhibit antibiotic‐resistant bacteria. For instance, Xu et al. (2018) transformed allicin isolated from garlic bulbs into nanometer‐sized iron sulfides. These nanoparticles can release bactericidal polysulfanes and have demonstrated proficiency in eradicating biofilm formations and exerting potent inhibition against antibiotic‐resistant strains of *S. aureus*. Similarly, isothiocyanates sourced from nasturtium (*Tropaeolum majus*) and horseradish (*Armoracia rusticana*) have antimicrobial potential against antibiotic‐resistant bacteria like *P. aeruginosa*, particularly in biofilm suppression (Kaiser et al., 2017). When combined with meropenem, isothiocyanates enhanced antibiotic efficacy by suppressing the metabolic activity of *P. aeruginosa* biofilms. In addition, da Silva et al. (2024) extracted six saponins—jujuboside B, jujubasaponin III, ziziphin, jujubasaponin IV, jujuboside II, and zizyphus saponin I—along with three saponin derivatives from *Sarcomphalus joazeiro*. The saponin‐enriched fraction showed a potentiating effect when used in combination with gentamicin and norfloxacin, increasing their activity against multidrug‐resistant *P. aeruginosa* and *S. aureus*. Saponins interact with the bacterial cell wall, causing structural damage, altering membrane permeability, and inducing membrane rupture.

Terpenoids have been shown to inhibit antibiotic‐resistant bacteria. Moo et al. (2021) found that 1,8‐cineol exhibited antimicrobial effects against carbapenem‐resistant *K. pneumoniae*. It increased the bacterial surface charge, enhanced outer membrane permeability, and induced oxidative stress, leading to membrane damage, leakage of intracellular components (such as nucleic acids, proteins, and lipids), and ultimately cell death. Another terpene, linalyl anthranilate (LNA), found in plants such as lavender and thyme, has demonstrated bactericidal activity against carbapenem‐resistant *K. pneumoniae*. Yang et al. (2021) demonstrated that LNA, when combined with meropenem, reduced MIC, making the bacteria more susceptible. The antimicrobial effect of LNA is attributed to its ability to induce oxidative stress by generating ROS, which could further initiate lipid peroxidation and cause damage to the bacterial membrane. Terpenes are also recognized as significant inhibitors of bacterial efflux pumps (Seukep et al., 2020). Oluwatuyi et al. (2004) identified several terpene compounds in *Rosmarinus officinalis*, such as carnosic acid, carnosol, and 12‐methoxy‐*trans*‐carnosic acid. These compounds have been shown to enhance the activity of erythromycin against multidrug‐resistant *S. aureus* by inhibiting the NorA efflux pump, thereby reducing the bacteria's ability to expel the antibiotic and increasing its intracellular concentration.

Alkaloids are also found to inhibit the antibiotic‐resistant bacteria. Guefack et al. (2022) reported that protoberberine alkaloids (columbamine, pseudocolumbamine, jatrorrhizine, palmatine, 4,13‐dihydroxy‐3,9,10‐trimethoxyprotoberberine, and 13‐hydroxy‐2,3,9,10‐tetramethoxyprotoberberine) identified from *Enantia chlorantha* could effectively restrain the growth of multidrug‐resistant bacteria including *E. coli*, *Enterobacter aerogenes*, *K. pneumoniae*, *Providencia stuartii*, and *S. aureus* with MIC values below 100 µg/mL. Importantly, one of these alkaloids, named columbamine, inhibited the H^+^‐ATPase pump and hindered the production of cellular ATP. Some alkaloids are also found to enhance the antibacterial effectiveness of antibiotics. Matrine derived from *Sophora alopecuroides* seeds was reported to display a synergistic effect with ciprofloxacin against antibiotic‐resistant strains of *P. aeruginosa* (Pourahmad Jaktaji & Koochaki, 2022). Total alkaloids demonstrated an impact on inhibiting biofilm formation and decreasing the activity of Mex pumps, thus enhancing bacterial susceptibility to fluoroquinolones. Additionally, Barbosa et al. (2021) isolated alkaloids from leaves of *Erythroxylum revolutum* Mart., including 6‐(2′‐methylbutyryloxy)‐3‐hydroxytropane and 6‐butyryloxy‐3‐hydroxytropane, which enhanced the effectiveness of norfloxacin and erythromycin against multidrug‐resistant *S. aureus*. This effect is likely linked to the ability of tropane alkaloids to impair ABC multidrug transporters in multidrug‐resistant *S. aureus*.

In summary, phytochemicals exert antimicrobial capacity by disrupting bacterial membranes, inhibiting efflux pumps, and impeding the formation of bacterial biofilms. When combined with antibiotics, some phytochemicals exhibit a synergistic effect that can enhance antibiotic effectiveness and modify antibiotic resistance, indicating the potential to manage antibiotic‐resistant bacteria in the gut and influence the gut resistome. In the future, it will be necessary to investigate the bioavailability, dosage, and long‐term effects of these phytochemicals in the human gut, which could lead to more targeted dietary interventions. Advancing our understanding of how specific phytochemicals can selectively modulate the gut resistome will be crucial for developing innovative approaches to mitigate the spread of resistance genes and improve human health outcomes.

#### 4.2.3 Probiotics

Probiotics, which are live microorganisms with health benefits, are commonly incorporated into fermented foods like yogurt and cheese (Fentie et al., 2024). However, it is important to note that these products typically contain probiotics only when made with unpasteurized milk, as pasteurization can significantly reduce or eliminate viable probiotic strains. Probiotics can influence antibiotic‐resistant bacteria in the gut by regulating the intestinal microenvironment, enhancing immune function, and inhibiting allergic reactions (Victoria Obayomi et al., 2024). Recent market trends indicate significant growth in the global probiotic industry. In 2023, the global financial revenue from probiotics reached 87.7 billion USD, reflecting growing demand for probiotic‐enriched functional foods and dietary supplements (Lei et al., 2025).

Certain probiotic strains have demonstrated antibacterial activity against resistant pathogens (Table 1). For example, *Lactobacillus acidophilus* NCFM and *Lacticaseibacillus rhamnosus* GG exhibited antibacterial effects against methicillin‐resistant *S. aureus* by disrupting the integrity of bacterial cell membrane (Nataraj et al., 2021). Additionally, encapsulated probiotics (*L. acidophilus* CL1285, *Lacticaseibacillus casei* LBC80R, and *L. rhamnosus* CLR2), when combined with tobramycin, exhibited a synergistic effect on methicillin‐resistant *S. aureus* and *P. aeruginosa* by restricting pathogen colonization and impeding the formation of biofilms (Li et al., 2018). Bifidobacteria are known to produce biosurfactants and acetate, which help in detaching adhering *E. coli* and altering the intracellular anionic composition (Victoria Obayomi et al., 2024). This can enhance the entry of tetracycline into *E. coli*, disrupting protein synthesis and ultimately resulting in the synergistic killing of *E. coli*. These studies pave the path for the potential application of probiotics as a functional food to regulate antimicrobial resistance in the gut.

In addition to these individual probiotic strains, probiotics found in fermented foods, such as yogurt and kimchi, have been shown to inhibit the growth of antibiotic‐resistant bacteria. *Lactobacillus parafarraginis* isolated from commercial yogurt exhibited antimicrobial activity against 14 multidrug‐resistant bacteria, including *E. coli*, *P. aeruginosa*, *A. baumannii*, *E. aerogenes*, *Proteus mirabilis*, and *K. pneumoniae*. The antimicrobial substance produced by this strain is likely a bacteriocin‐like peptide or a protein, which is active against closely related bacterial strains, suggesting its potential role in inhibiting the growth of pathogenic multidrug‐resistant bacteria (Gomez‐Gallego et al., 2018). Yi and Kim (2023) isolated probiotic lactic acid bacteria (*Pediococcus inopinatus* K35) from kimchi, which could effectively decrease the growth and biofilm formation of multidrug‐resistant *P. aeruginosa*. These studies collectively highlight the promising potential of probiotics, especially those derived from fermented foods, as natural agents capable of combating antibiotic‐resistant bacteria.

However, the introduction of live organisms into the host through probiotic intake raises concerns about potential risks. One concern is the possibility that probiotic strains may carry ARGs, which can be transferred to pathogenic bacteria, thus contributing to the spread of resistance (Newman & Arshad, 2020). For instance, Selvin et al. (2020) isolated probiotic strains from dietary supplements and found penicillin G resistance in *Enterococcus faecalis* and *Bacillus mesentericus* and ampicillin resistance in *L. acidophilus*.

Additionally, a study by Montassier et al. (2021) showed that the intake of commercially available probiotic supplements (containing 11 common probiotic strains: *L. acidophilus*, *L. casei*, *Lacticaseibacillus paracasei*, *L. rhamnosus*, *Lactiplantibacillus plantarum*, *Bifidobacterium bifidum*, *Bifidobacterium breve*, *Bifidobacterium longum* subsp. *longum*, *Bifidobacterium longum* subsp. *infantis*, *Lactococcus lactis*, and *Streptococcus thermophilus*) led to a decrease in the abundance of ARGs. However, it was also observed that probiotics could act as a reservoir for the expansion of the gut resistome, as probiotic intake could expand ARG‐carrying strains to pathogens through horizontal gene transfer, as the detection of mobile genetic element contents (transposases and integrases) was correlated to ARG abundance.

These findings emphasize the need for caution when incorporating probiotics into the diet, especially in the context of commercial probiotic supplements. While probiotics can play a role in reducing the colonization of pathogenic bacteria and enhancing antimicrobial activity, their role as carriers of ARGs must be carefully assessed. To mitigate potential risks, it is essential to monitor the ARG content in probiotic strains used in supplements and functional foods, ensuring that they do not contribute to the further spread of resistance.

In conclusion, while probiotics offer potential as functional foods to modulate the gut resistome and combat antibiotic resistance, their potential to harbor and transfer ARGs calls for a more thorough investigation. Future research should focus on identifying probiotic strains with minimal risk of ARG transfer and on optimizing probiotic foods to enhance their therapeutic benefits while limiting adverse effects on the gut resistome.

## 5 CONCLUSIONS AND FUTURE PERSPECTIVES

Antibiotics play a pivotal role in combating bacterial infections. Nonetheless, the excessive utilization of antibiotics has expedited antibiotic resistance, resulting in the development of antibiotic resistome. ARGs can accumulate in natural environments through various anthropogenic activities, including the disposal of pharmaceutical waste and agricultural runoff. Antibiotic resistome can transfer to the human gut through the food chain and public exposure. Antibiotic resistance in the gut microbiome can accumulate through both vertical inheritance and horizontal gene transfer, while horizontal gene transfer allows bacteria to quickly adapt to antibiotic exposure by acquiring resistance genes from other bacteria, making it challenging to combat infectious diseases. Thus, the more rational and effective implementation of regulatory measures is crucial to managing antibiotic resistance and ensuring responsible antibiotic usage. Moving forward, it is imperative to develop antibiotics that are precisely targeted at distinct bacterial strains while concurrently opting for appropriate antibiotics to curtail the risk of antibiotic resistance and minimize inadvertent harm to beneficial microorganisms.

The gut microbiota plays a crucial role in shaping human health and influencing the development of various diseases. Research suggests a link between the gut resistome and various diseases, including metabolism disorders, cardiovascular disease, liver disease, and nervous system disease. However, the precise mechanism by which ARGs contribute to these diseases remains largely unknown. The current hypothesis is that ARG may alter the balance of the gut microbiota and influence host immune responses. Future research should focus on elucidating these mechanisms, particularly how ARGs interact with other microbial and disease outcomes, and exploring interventions that can selectively target antibiotic‐resistant bacteria or inhibit the spread of ARGs, helping to restore a healthier balance in the gut microbiota and mitigate the impact of gut resistome on human health.

Application of appropriate dietary strategies holds promise in tackling the challenge of antibiotic resistance. Various dietary components, including macronutrients, phytochemicals, and probiotics present in food, have demonstrated efficacy in inhibiting antibiotic‐resistant bacteria by interfering with bacterial membranes, hindering the development of bacterial biofilms, and potentially restricting the horizontal transfer of ARGs. However, it is noteworthy that certain probiotics might carry their antibiotic‐resistant genes, introducing potential side effects. In the future, personalized dietary recommendations tailored to an individual's gut microbiota composition and resistome profile, along with the development of functional foods enriched with targeted nutrients, could impact the gut microbiota and inhibit the horizontal transfer of ARGs to foster a healthier resistome composition.

## AUTHOR CONTRIBUTIONS

**Ze Liang**: Conceptualization; writing—original draft; visualization. **Zijian Liang**: Writing—review and editing. **Hang‐Wei Hu**: Writing—review and editing. **Kate Howell**: Writing—review and editing. **Zhongxiang Fang**: Writing—review and editing. **Pangzhen Zhang**: Conceptualization; writing—review and editing; supervision.

## CONFLICT OF INTEREST STATEMENT

The authors declare no conflicts of interest.
